# Online Health Information-Seeking Behaviour among People of African Descent in the United Kingdom: A Qualitative Study

**DOI:** 10.3390/healthcare12090897

**Published:** 2024-04-26

**Authors:** Jesse Enebi Usman, Charmaine Childs, David Rogerson, Markos Klonizakis

**Affiliations:** 1 Lifestyle Exercise and Nutrition Improvement (LENI) Research Group, Department of Nursing and Midwifery, Sheffield Hallam University, Sheffield S10 2BP, UK; 2 College of Health, Wellbeing and Lifesciences, Sheffield Hallam University, Sheffield S10 2BP, UK; 3 Sports and Physical Activity Research Centre, Sheffield Hallam University, Sheffield S10 2BP, UK

**Keywords:** cultural sensitivity, culturally tailored interventions, online health, internet-based intervention, people of African descent, health information-seeking behaviour

## Abstract

Effective public health interventions rely on understanding how individuals access, interpret, and utilise health information. Studying the health information-seeking behaviour (HISB) of a community can provide valuable insights to inform strategies that address community health needs and challenges. This study explored the online HISBs of People of African Descent (PoAD) in the United Kingdom (UK), a demographic that comprises four percent of the UK population and has a 92.8% active Internet usage rate. Data on the HISB were collected from 21 PoAD across various UK regions through online semi-structured interviews before being analysed using reflexive Thematic Analysis (TA). The participants ranged in age from 20 to 70 years and had a mean age of 42.8 (SD ± 11.4). Our analysis of the interview transcripts revealed five key themes: Internet usage and preferences, attitudes toward social media, barriers to seeking health information online, trust in online health information, and cultural influences on online HISB. Our findings indicate a proactive engagement among PoAD in seeking health information online that is underscored by a preference for professional sources over ethnic congruence. However, concerns about misinformation exist, and there are barriers to accessing health information online, including data privacy, unreliable information, and information relevance and overload. We also found that cultural factors and traditional beliefs impact the adoption of Internet-based interventions among PoAD, highlighting the need for culturally sensitive approaches. Preferences regarding the frequency and delivery of online health information varied among participants, with a majority preferring a weekly update. This study emphasises the critical need for accessible, culturally appropriate, secure, and reliable online health resources tailored to the needs and preferences of the PoAD.

## 1. Introduction

The advent of the Internet has revolutionised global access to health information, making it a pivotal tool in public health and individual health management. As a versatile platform for sharing information and facilitating social interaction, the Internet’s role in health-related pursuits has grown exponentially [1,2]. The potential for digital platforms to serve as a primary source of health information is significant, as Internet access in homes in the United Kingdom (UK) is nearly universal, with most of the population utilising this resource to search for health information [3]. This digital transformation presents a novel environment where healthcare providers and patients can efficiently exchange health information [4]. Data from the Office for National Statistics (ONS) in 2020 indicated that 96% of households in the UK had Internet access, with more than half using it to seek health information [3,5]. People of African descent (PoAD) comprise four percent of the UK population, with 92.8% actively using the Internet [5]. The ease of accessing a wide array of information has made the Internet a valuable resource for healthcare providers and patients [4]. The proliferation of information and communication technologies, including social media, has facilitated the widespread use of the Internet for seeking health information [6,7], encouraging individuals to turn to online resources for health support [8,9].

Health Information-Seeking Behaviour (HISB) is a dynamic process that empowers individuals to seek health information, directly influencing their health-related decisions and outcomes [10,11]. Motivated by a spectrum of needs, from addressing specific health conditions to assessing potential risks and benefits, HISB is instrumental in shaping health literacy and management [12]. HISB is an active need-fulfilment behaviour where individuals obtain health information from various sources [13]. It is particularly relevant in the digital age, where the Internet has become a primary medium for HISB, providing timely and cost-effective access to health information [14]. However, using the Internet to seek health information has its challenges, as the potential for information overload and the risk of misinformation pose significant concerns that can lead to poor health decisions [15]. As the Internet’s role in HISB grows, understanding the nuances of how different communities engage with online health resources becomes increasingly important.

Ethnic minorities, including PoAD, encounter various forms of stigma, racism, and discrimination. These challenges amplify their vulnerability to various disease-risk factors and may restrict their access to high-quality health services, leading to poor health outcomes [16]. This situation was especially pronounced during the COVID-19 pandemic, underscoring the critical need for access to comprehensive and culturally appropriate health services and information [17]. Overcoming the barriers to health information access is essential for improving health outcomes and achieving equity. The Internet provides a unique opportunity for addressing these barriers by breaking down traditional fences to information and enabling tailored health interventions. Despite the potential of the Internet to bridge information gaps and improve health outcomes, there is a lack of research focusing on the online HISBs of PoAD in the UK. This study fills this gap by exploring how PoAD in the UK seek and utilise health information online. This research is essential for advancing our understanding of how digital platforms can be leveraged to enhance health equity and promote the wellbeing of ethnically diverse populations. This study can also inform the development of tailored interventions that meet this population’s unique cultural and healthcare needs.

## 2. Methods

### 2.1. Sampling and Recruitment Process

We recruited UK residents of African and Afro-Caribbean descent who are 18 or older and proficient in navigating Internet-based devices. This study defines PoAD as a group consisting of individuals of African and Caribbean descent. Participants were selected through purposive sampling, a research method that involves choosing individuals with specific characteristics and qualities of interest [18]. Patton [19] describes purposive sampling as a technique that enables researchers to select case studies that offer relevant information about the phenomenon of interest, making it ideal for qualitative studies that focus on complex behaviours and perspectives within a cultural context. Consequently, the sampling criteria in this study were not intended to ensure randomness of the sample, but rather to capture a wide range of perspectives by including individuals with diverse backgrounds in terms of age, gender, and geographical location within the UK. 

Recruitment channels included word-of-mouth, email invitations, and social media platforms, ensuring that only eligible individuals were recruited. All participants were required to provide written informed consent, indicating that they understood the study’s objectives and were willing to participate of their own accord.

### 2.2. Eligibility Requirements

To participate in this study, individuals had to meet several requirements to ensure that they were capable of contributing valuable insights. Participants had to be UK residents of African or Afro-Caribbean descent, aged 18 years or older, who were able to read and write in English. It was essential for participants to be generally healthy and have no chronic illnesses that could hinder their ability to participate effectively in the study. Furthermore, all participants were required to have sufficient cognitive ability to provide informed consent and engage in the study activities. Finally, proficiency in navigating Internet-based devices was also a requirement to ensure that all participants could actively participate in the online semi-structured interviews.

### 2.3. Ethics Approval

This study was approved by Sheffield Hallam University’s Ethical Committee (Ethic Review ID: ER45058101). The study’s ethical considerations include ensuring voluntary participation, protecting participant rights, and maintaining confidentiality.

### 2.4. Data Collection 

JEU conducted 21 interviews online through Zoom^®^ (version 5.12.3) [20,21] over a period of 8 weeks, from October to December 2022. Each interview lasted between 30 to 60 min. Before conducting the interviews, JEU developed an interview guide based on a comprehensive literature review and preliminary consultations with PoAD. The guide included topics such as sources of health information, devices used to access health information online, trust in online content and professionals, and the impact of cultural beliefs on online HISB. The interviews were conducted in a conversational style to promote in-depth conversations with the participants. The interviews were recorded and transcribed verbatim for further analysis [22].

### 2.5. Data Analysis

We utilised reflexive Thematic Analysis (TA), as defined by Braun and Clarke [23], to investigate the online HISBs of PoAD in the UK. We chose reflexive TA due to its flexibility and depth in examining intricate phenomena within specific cultural contexts [23]. Reflexive TA offers a nuanced understanding of the experiences of the participants by prioritising the co-creation of knowledge between researchers and participants [24]. When compared to other qualitative analysis approaches, our study’s constructionist epistemology makes reflexive TA a suitable method [25].

According to Braun and Clarke [23,24,26], it is essential to establish various theoretical assumptions across various continua to guide studies employing reflexive TA. One such continuum is essentialist versus constructionist epistemologies. This study adopts a constructionist epistemology [25], which considers knowledge to be co-constructed between the researcher and participants. This approach is essential for exploring the online HISB within the cultural context of PoAD in the UK. The second continuum is experiential versus critical orientation to data. Our study adopted an experiential approach [25] to capture participants’ experiences and perceptions. This approach is pivotal to understanding the online HISBs of PoAD. The third continuum is inductive versus deductive analysis. An inductive approach was adopted in this study to allow themes to emerge organically from the data [25]. Moreso, this approach helps us avoid imposing preconceived views on the data and facilitates the discovery of unanticipated insights. Lastly, we employed both semantic and latent coding continuum [25]. By using this dual coding strategy, we were able to examine the online HISBs of the participants thoroughly while capturing both the overt and subtle nuances in their narratives.

We followed the six-phase framework of reflexive TA detailed by Braun and Clarke [24,26] and explained in Byrne [25]. JEU analysed all interviews, identifying initial codes through repeated readings. The initial codes were grouped into potential themes, which were then refined and validated to ensure they accurately represented the data and captured the online HISBs of the participants. The refined themes were then integrated into the analytical report [25]. Reflexive TA does not prioritise the positivist notion of ‘coding reliability’ that is typical of other qualitative methods; therefore, it is acceptable for one researcher to code and analyse the data [23,27]. The replication of codes or themes by different researchers is not an expectation, as reflexive TA values the unique interpretations that individual researchers bring to the data [25]. This feature of reflexive TA is not a limitation but rather a strength, enhancing the depth and authenticity of qualitative research by valuing the richness and complexities of human experiences and the subjective meanings attached to them.

Reflexivity was fundamental in this study to ensure that findings were grounded in data and not skewed by the researcher’s biases. JEU’s background in public health and mental health nursing, coupled with being an individual of African descent, enriched the analysis by providing deep cultural insights. Throughout the research, JEU engaged in reflexive practices, including maintaining a reflexive journal and discussions with the co-authors to challenge and refine the analysis process. These steps helped to mitigate any potential biases and ensured that the analysis remained true to the participants’ perspectives and experiences.

### 2.6. Sample Size Determination

Due to the interaction between epistemological, methodological, and practical factors, the determination of the required number of participants in a qualitative study is considered complex by many researchers [28]. Reflexive TA necessitates a departure from traditional concepts of data saturation towards a more nuanced understanding of information power and data adequacy. Data saturation refers to the point at which no new information or themes emerge from the data [29,30]. The applicability of data saturation to reflexive TA has come under scrutiny due to the central role the researcher plays in the analysis of the data [31]. In reflexive TA, the meaning of data is not static; rather, it emerges from the active interpretation of the researcher, implying that new insights can continue to emerge with ongoing data analysis [32]. Therefore, establishing a concrete point of saturation is challenging due to the interpretative nature of reflexive TA, which is influenced by the researcher’s theoretical lens, the depth of the data, and the diversity of the sample [33].

In this study, we made a reflexive judgment on the sample size based on the principle of information power and the goals of our analysis (rather than aiming for data saturation). The principle of information power suggests that the more relevant information a sample holds concerning the study aim, the smaller the sample size required [33]. This approach acknowledges the pragmatic nature of qualitative research, where practical considerations such as time and resources can affect the analytical depth and breadth required to address the research question [34,35]. The shift in perspective reflects a broader understanding of sample size determination in qualitative research that is more aligned with the dynamic and interpretative nature of reflexive TA [31,33].

## 3. Results

### 3.1. Demographic Characteristics of Respondents 

This study collected demographic data from 21 participants (as shown in Table 1), with 52% (11/21) identifying as Africans and 48% (10/21) as Afro-Caribbeans. This provided valuable cultural insights into the study’s findings. The group was composed of 67% females (14 out of 21) and 33% males (7 out of 21). The ages of the participants varied from 20 to 70 years old, with a mean of 42.8 (SD ± 11.4). We purposefully selected a broad age range to ensure a comprehensive understanding of the online HISBs of PoAD across different life stages and experiences. All participants used smartphones. Other devices utilised to access the Internet included laptop computers (42%), desktop computers (38%), tablets (33%), wearables (33%), and smart TVs (29%). We recruited participants from a diverse range of urban and suburban areas in the UK, including Manchester, Cardiff (Wales), Belfast (Northern Ireland), Leeds, Sheffield, London, Glasgow (Scotland), and Birmingham. This diversity in the sample allowed us to explore the HISBs of PoAD from different perspectives and provided a balanced mix of participants.

### 3.2. Participant Selection

**Figure 1:**
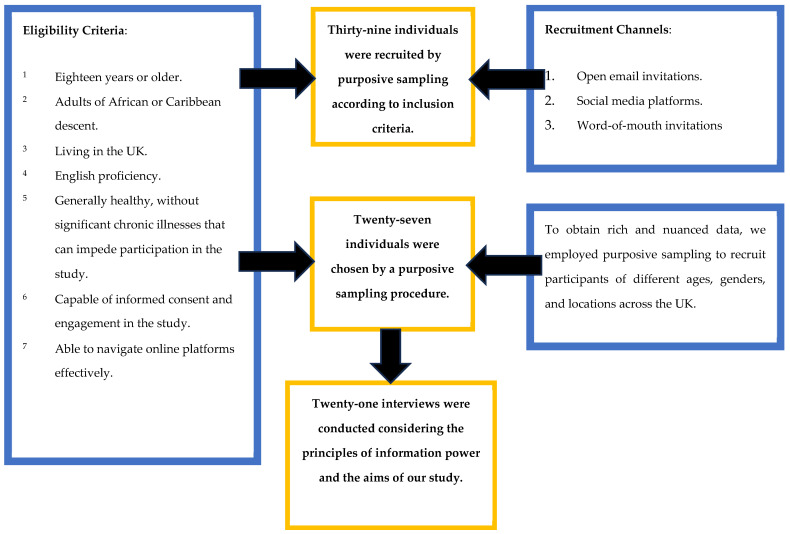
Eligibility Criteria, Recruitment Strategies and Participant Selection.

We initially recruited 39 participants who responded to our advertisements and met our inclusion criteria. After the initial recruitment, we selected 27 participants based on their potential to provide diverse and nuanced insights, as outlined in the participant selection flowchart (Figure 1). The strategy was crucial to ensuring that our study benefited from a wide range of information and perspectives, thus enhancing the depth and relevance of our findings [18].

### 3.3. Identified Themes

Five overarching themes emerged that represented the participants’ online HISBs. 

#### 3.3.1. Internet Usage and Preferences

All participants acknowledged the Internet’s significance as an invaluable resource for accessing information, emphasising its role in enabling communication without geographical barriers. Participant 7 (male, 49), highlighted the Internet’s potential in bridging distances, stating, “*…it is a vehicle through which we reach people irrespective of their locations… We can connect and have a conversation…*” Moreover, some participants acknowledged the transformative impact of the Internet, highlighting its ability to foster global connectivity and its potential for social networking, cross-cultural communication, and global transactions. Participant 19 (female, 60) expressed this sentiment: “*…It is the tool that makes the globe a village… all these connections on social media… communication with families and friends around the globe… you can make transactions globally…*” Additionally, some participants regarded the Internet as an “equality tool”, acknowledging its capacity to democratise access to information and create equal opportunities. Participant 18 (female, 43) highlighted this aspect: “*… It is a great tool that makes data available to everyone. You can call it an equality tool*”.

Most participants (15/21) responded positively to seeking health information online, recognising the benefits of accessing it online and particularly emphasising its low cost and convenience. Participant 10 (female, 70) stated, “… *I think the use of Internet search for information online one is very convenient, its cost-effective, from the comfort of your house you can just surf the Internet to get information on how to live healthy, and get more information about your health, and who knows sometimes you might get solution to some of your problems*”. Conversely, some participants (6/21) expressed conservatism regarding Internet use. Participant 19 (female, 60) stressed the importance of explicit consent and verified information, saying, *“Not very safe. … it’s not clear what will happen to my personal data. Also, it is difficult to identify which site presents the authenticated information”.*

Several participants preferred Internet interventions that connect health information seekers with personalised sources rather than relying solely on generic online resources. They believed such platforms would be more effective in meeting individual needs. Participant 16 (female, 45) stated, “*There is plenty of information online, but I would also say there is a lot of room for improvement. If there are apps or sites that connect patients to the healthcare professionals, that would be better*”. Participant 14 (female, 40) echoed this sentiment, suggesting that connecting with healthcare providers through online platforms could yield more effective outcomes than simply searching symptoms on search engines: “*…connecting with healthcare providers and their platform can be more effective than just searching symptoms on Google*”. Similarly, Participant 13 (male, 20) emphasised the appropriate usage of online health information and highlighted the efficiency of contacting health professionals directly: “*It is a good stuff and people should use it appropriately. If someone can get in contact with health professional, it can be more efficient*”.

Regarding the frequency of receiving health information, the respondents demonstrated varied preferences. Four participants preferred daily updates, while three preferred receiving information a few times a month. Over half of the respondents (14/21) wanted to receive health information online weekly. However, some participants highlighted that excess information could hinder their willingness to access health information online. Participant 1 (female, 27) stated, “*About once a week… It gives me time to carry out more research and analysis on the information*”. Similarly, Participant 17 (male, 36) preferred receiving information once a week, citing the annoyance caused by sources that inundate users with overwhelming daily information. The participant stated, “*Perhaps once a week. Some websites share overwhelming information daily, making it annoying*”. Contrastingly, Participant 10 (female, 70) indicated a preference for receiving health information daily, stating, “*On a daily basis. I don’t mind*”. Similarly, Participant 3 (female, 34) stated, “*One a day is fine. Some websites just keep sending you annoying information, I don’t like that*”. Participant 2 (female, 39) wanted to receive health information monthly, stating, “*I would like to receive health information monthly*”. Participant 18 (female, 43) stated, “*The type of information matters, I would be okay with a few times in a month*”, indicating a preference for receiving health information multiple times within a month.

Additionally, some participants preferred receiving summarised information without technical jargon to enhance comprehension. Participant 5 (female, 37) highlighted the importance of concise and straightforward language, stating, “*The information has to be concise, simple English. Not too much jargon or anything. If you like use bullet points*”.

#### 3.3.2. Attitude towards Social Media

The participants’ perception of social media as a means of connecting people and facilitating the exchange of ideas emerged as a prominent theme. A majority of the participants (14/21) acknowledged social media as a communication and idea-sharing tool. Furthermore, nearly half of the participants (10/21) identified social media as a platform for accessing and disseminating information. Participant 1 (female, 27) stated, “*I think it is where people meet to talk and exchange ideas*”, emphasising the interactive nature of social media. Similarly, Participant 14 (female, 40) described it as “*an avenue for interaction, people meet and share information and also exchange ideas*”, highlighting its role in fostering social connections. Moreover, some participants recognised social media’s global reach and potential for information exchange across geographical boundaries. Participant 8 (male, 51) expressed this perspective, stating, “*…social media is a global village where the exchange of information takes place from any part of the world*”. Additionally, Participant 13 (male, 20) emphasised the speed and effectiveness of information dissemination through social media, stating, “*Anything posted there can reach people more quickly, and this would contribute to better information dissemination*”.

Some participants regarded social media as a means of entertainment that aids in managing daily tasks. Participant 2 (female, 39) expressed her enjoyment of social media, stating, “*I like social media because it keeps me entertained*”. Similarly, Participant 5 (female, 37) described their frequent engagement with Facebook as a source of enjoyment, stating, “*I am on Facebook a few times daily, and it is a good distraction and fun*”.

On the other hand, a few participants viewed social media as a potential distraction from essential responsibilities. Participant 3 (female, 34) said, “*I love social media sometimes, but it can also be a distraction*”, indicating the need for a balance in its usage. Another participant cautioned against the spread of inaccurate information on social media platforms, highlighting the need for caution and critical evaluation of information, stating, “*care must be taken while on social media because wrong information can also be passed*” (Participant 1, female, 27). 

Fifteen participants had Facebook accounts, 11 of whom were comfortable receiving health-related information through the platform. Participant 2 (female, 39) indicated their preference, stating, “*…if it is general information, I can do Facebook…*”. Similarly, participant 14 (female, 40) expressed their satisfaction with Facebook, stating, “*I like Facebook, it is where I can find most of the people*”.

All participants had WhatsApp accounts, although only four respondents expressed willingness to receive health information through this platform. Other social media platforms mentioned by participants included Twitter, Instagram, and LinkedIn. Participant 21 (male, 49) shared their preference for receiving health information via WhatsApp and Facebook, stating, “*I like WhatsApp and Facebook. Those are where most of my friends are, and I enjoy being there. I get there a few minutes every day*”. Participant 11 (male, 51) also emphasised their reliance on Facebook and WhatsApp, stating, “*I’m not a very sociable person, but for me Facebook and WhatsApp are enough*”. Participant 2 (female, 39) expressed flexibility in their preferred platforms, stating, “*I can do Facebook or Instagram*”.

Email was highlighted explicitly in a smaller subset of respondents as their preferred platform for receiving health-related information. Participant 5 (female, 37) noted, “*Probably* via *email if it is personal information but I don’t mind receiving public health information on social media*”.

#### 3.3.3. Barriers to Seeking Health Information Online

The participants identified several barriers to seeking health information online. Privacy concerns emerged as a prominent deterrent, with respondents expressing apprehension about the potential exposure of personal data during online health searches, “*I do not like my information being exposed, like private health information. Okay, what if there is a leak in the system?*” (Participant 1, female, 27). 

Another barrier identified by participants was the consideration of topical relevance while seeking online health interventions. Participant 5 (female, 37) emphasised the importance of obtaining information that is personally relevant and understandable, stating, “*What can stop me is if the information I’m getting is not relevant to me, or if it’s not of use to me, or it’s just something I don’t understand*”.

Furthermore, information overload was cited by two respondents as a hindrance to seeking health information online. Participant 8 (male, 51) expressed the difficulty of obtaining the right information from the vast array available online, stating, “*What can hinder me from searching for health information online is too much information. Especially an inability to get the right information from the wide range of information we have on the Internet*”. Similarly, Participant 6 (female, 51) highlighted the overwhelming frequency of information, stating, “*…if the frequency of the information is overbearing, I just unsubscribe*”.

A lack of or unreliable Internet connections emerged as another significant barrier, with some participants indicating that limited access to the Internet can prevent them from seeking health information online. Participant 10 (female, 70) identified geographical location as a factor, stating, “*I’d say geographical location, for example, if I am in a place where I can’t access the Internet, I won’t be able to access any information online…*”. Participant 13 (male, 20) stressed the importance of Internet access for information dissemination, stating, “*The access to Internet tools is very important. So, the target group should be supported to have for example good Internet connections and devices*”.

Participants highlighted inaccurate or misleading information as a significant barrier to seeking health information online, expressing a desire for accurate and reliable sources of information. Participant 3 (female, 34) stated, “*Misinformation. I stop using them if they stop giving me accurate information*”. Similarly, Participant 17 (male, 36) expressed the need for accurate information, stating, “*If the info I am getting is incorrect, I would have to stop*”. The importance of authenticating sources was also highlighted, as Participant 13 (male, 20) mentioned, “*This is without forgetting that some sources might need to be authenticated…*”. Additionally, Participant 8 (male, 51) mentioned the challenge of discerning reliable information from various websites, stating, “*I feel there’s a mixture there because you might be searching for information and might be left with a lot of websites to choose from, which makes it difficult because some information you find on some websites may not be genuine, or sufficient. The ability to get the right information from the right website is one of the challenges in using the Internet to access information*”.

#### 3.3.4. Trust and Reliability of Online Information

All respondents acknowledged that they trust health information provided by health professionals irrespective of their ethnicity. Participant 3 (female, 34) emphasised the importance of professional expertise, stating, “*…as long as the person is a professional in the field. I do not have ethnicity preference*”. Similarly, Participant 4 (male, 54) stated, “*I would say a professional in that field. So, depending on the information required, I’d prefer a professional in that field to deliver such information to me. Ethnicity does not matter, as long as the person is a professional*”.

#### 3.3.5. Cultural Influences on Online HISB

Awareness and use of Internet-based interventions (IbIs) among PoAD was limited, necessitating the need to increase awareness of online health interventions within this community. Participant 3 (female, 34) stated, “*Yes, so for the Black community we need awareness. We need to take responsibilities for our own health… They tell me they have prayed about it. They need awareness not only to access the apps or websites but take their health issues more seriously*”. Furthermore, Participant 20 (female, 42) expressed that some PoAD refuse to use IbIs due to a lack of trust in online platforms. They acknowledged the conservative nature of certain African cultures and the scepticism towards online sources for health information, stating, “*Yeah, Blacks are not quick in trusting online stuff as compared to whites. Personally, I am okay searching general information online, but I am not ready to share personal information. We are conservative, you know*” (Participant 20, female, 42).

Moreover, one participant highlighted the influence of traditional beliefs among some PoAD, leading them to dismiss the need for IbIs. They mentioned the persistence of African traditional healing practices and the inclination to seek assistance from native doctors. Participant 2 (female, 39) shared their perspective, stating, “*There are some people who still practice or who still believe in a lot of African ways of treating diseases… some people they use some different means to make sense of their situation like using different things like trees or wood…*”.

## 4. Discussion

This study explored the online HISBs of PoAD in the UK. The study also sought to identify the obstacles these individuals face when searching for health-related information online, aiming to suggest potential solutions to overcome these challenges. This section presents a comprehensive analysis of the study’s findings, incorporating perspectives from the relevant academic literature. We also present the implications of our findings for public health policy, suggesting that a more inclusive approach to digital health information may help to mitigate health disparities.

### 4.1. Gender Distribution and Online HISB

We noted a gender distribution leaning towards female participants, with a ratio of approximately 1:2 (male: female), indicating a higher response rate from females to our research participation invitation. This gender imbalance could be due to the increased readiness of women to participate in research [36,37]. The reasons for this difference are multifaceted and may include women having a higher awareness of health [38] or a better understanding of the relevance of research. Our research highlights that gender can be a significant determinant of HISB and health outcomes, reflecting the influence of societal norms, personal beliefs, and the perceived severity of health issues [39,40]. Previous studies have shown that men tend to seek more professional online health information, while women are more likely to seek emotional support [40]. According to Rowley et al. [39], women tend to consult more types of sources than men, while men are more likely to search for information regarding long-standing health conditions. This study did not reveal significant differences in HISB between male and female participants, suggesting that gender may not be as critical a determinant of HISB in this population. We acknowledge that the gender ratio in our study may have influenced these findings, potentially obscuring gender-specific nuances in HISBs of PoAD. This limitation highlights the need for further research to explore gender dynamics in HISB among PoAD, ensuring a balanced representation of both genders to accurately capture and compare their HISB.

Our findings underscore the importance of considering cultural identity, social determinants of health, and individual experiences over gender when designing health information interventions for PoAD. Future research should also investigate the interplay between gender, culture, and social determinants of health in shaping HISB among PoAD. The findings of such research could offer new insights that could inform the design of more inclusive, accessible, adaptable, and culturally sensitive health campaigns and interventions for the PoAD.

### 4.2. Ethnic Backgrounds and Online HISB

Ethnicity, past experiences, and health beliefs can affect online HISBs [41]. This study compared the online HISBs of African and Afro-Caribbean participants to investigate the potential influence of shared cultural practices on online HISBs. We found that there was a similarity between the online HISBs of African and Afro-Caribbeans, suggesting that cultural practices and beliefs that are shared between communities could be more influential in shaping HISBs than distinct ethnic practices. This finding underscores the importance of considering the broader cultural context that encompasses both African and Afro-Caribbean communities when designing health interventions and promoting healthy behaviours. However, it is vital to acknowledge the complexity of cultural identity and its potential influence on health behaviours. Although our study provides a basic understanding of the similarities in the online HISB between African and Afro-Caribbean participants, it also underscores the need for further research to explore the nuances and differences between these groups.

### 4.3. Cultural Influence on Online HISB

The cultural context of PoAD significantly influences their online HISB, shaping their preferences, trust, and engagement with online health resources. Cultural beliefs and practices influence how health information is sought, interpreted, and utilised [17]. Some participants expressed concerns about data privacy and misinformation, worsened by cultural perceptions of the Internet as an impersonal and potentially unreliable source of health information. Our findings indicate that cultural identity and traditional beliefs among PoAD impact their receptiveness to IbIs, while the scepticism towards online health information sources, as emphasised by some participants, can be a result of a broader mistrust rooted in historical experiences of discrimination and marginalisation in healthcare settings [16]. Moreover, the preference for health information delivered by qualified healthcare professionals, irrespective of ethnicity, underscores a universal trust in professional expertise over the source’s cultural alignment. This finding challenges the assumption that ethnic congruence between the information provider and seeker is paramount, suggesting that professional credibility goes beyond cultural boundaries in the context of online HISB among PoAD. Furthermore, the influence of traditional beliefs on the adoption of IbIs among some PoAD indicates a complex relationship between modern healthcare practices and traditional healing methods. IbIs must be developed with a deep understanding of these cultural nuances to effectively engage PoAD. The relevance, adoption and effectiveness of online health information can be enhanced by considering the unique cultural, social, and historical contexts of the target population [42]. Incorporating culturally sensitive communication styles, narratives, and health education materials can bridge the cultural gap, fostering a more inclusive and accessible digital health environment for PoAD. 

Further investigation is needed to fully understand the influence of cultural beliefs and practices on the online HISB among PoAD. For instance, the reliance of some PoAD on traditional healing practices and the scepticism towards online health information sources reflect broader cultural narratives that can shape health behaviours. According to Betancourt et al. [42], cultural competency involves understanding and responding to cultural differences in health beliefs, practices, and values. Healthcare providers and policymakers can improve online HISB among PoAD and ultimately contribute to reducing healthcare disparities by taking a culturally responsive approach to designing health information systems.

### 4.4. Internet Access and Usability

The findings of this study underscore a significant reliance on smartphones among participants for accessing the Internet, reflecting a broader societal shift towards mobile connectivity and the increasing dependence on Internet-based devices for information retrieval. This trend is in line with global movements towards digital and mobile platforms as primary sources of information access [43,44]. The pronounced preference for smartphones in the online HISBs of PoAD in the UK stresses a need for health information resources that are optimised for mobile use. To effectively meet the needs of this demographic, it is necessary to develop health information resources while adopting a mobile-first approach, which includes implementing responsive web designs that ensure content is easily accessible across a range of mobile devices, simplified navigation to enhance the user experience, and incorporating interactive features that engage users and facilitate a more dynamic interaction with health information [45]. These design considerations are not just enhancements but necessities in ensuring that health information is accessible, understandable, and usable for PoAD.

Furthermore, the participants reported using other digital devices beyond smartphones, including laptops, desktop computers, tablets, wearables, and smart televisions, to access the Internet. This diversity in digital access points highlights the importance of developing versatile and adaptable health information resources that are compatible with a wide range of devices. Ease of access and a seamless user experience must be ensured across different platforms to effectively cater to this community’s varied needs and preferences. 

### 4.5. Frequency and Preferences for Online Health Information

Demirci et al. [43] found that regular Internet users seek health information more frequently. This study enriches this understanding by revealing that a significant majority (over 65%) of the participants prefer to receive health information weekly. The preference for regular updates underscores a proactive engagement with health information and reflects an ongoing need for current and relevant health information. The participants emphasise the accuracy, currency, simplicity, privacy, and security of online health information, pointing to a discerning approach to health information consumption. This discernment indicates that PoAD value not just the information but also its quality and reliability. 

The expressed preference for information provided by certified and suitably qualified healthcare professionals further underscores a trust in professional authority irrespective of these professionals’ racial or cultural backgrounds. This finding is particularly significant, as it suggests openness among PoAD in regard to diverse healthcare advice and information sources, challenging any notions of homogeneity in health information preferences within ethnic groups. The findings also indicate that the frequency and nature of health information sought online by PoAD are influenced by individual preferences, cultural nuances, and the broader digital health-information landscape. These insights offer valuable implications for healthcare providers and policymakers in tailoring online health information strategies that are not only accessible but also resonate with the cultural and informational needs of PoAD. 

### 4.6. Role of Social Media in Health Information Seeking

Social media platforms offer numerous benefits, including disease surveillance, communication, and health education [46,47]. However, participants in this study expressed concerns about the accuracy of the information on these platforms and the potential for spreading misinformation, echoing the findings of Oyeyemi et al. [48]. The study reveals a preference among participants for social media platforms that combine visual appeal with ease of use, indicating the importance of design and accessibility in attracting users seeking health information online. This preference suggests a potential research area focusing on how design elements can effectively facilitate health information dissemination on social media.

Notably, the study underscores the role of social media, particularly Facebook, as a key source of health information. This finding mirrors those of Maon et al. [7], highlighting social media’s role in health information dissemination. Consistent with Augustaitis et al. [49], the participants of this study highlighted the necessity of content moderation, privacy, and security measures in regard to obtaining health information online. 

### 4.7. Perceived Barriers to Seeking Health Information Online and Possible Solutions

As noted by Xuexia et al. [50], certain participants highlighted insufficient health literacy as a considerable obstacle to accessing online health information, underscoring the necessity for public health education campaigns to enhance general health literacy. To encourage people to seek health information online, governments and healthcare organisations can implement initiatives that incentivise such behaviour [45]. Furthermore, health information dissemination should align with users’ media consumption habits by leveraging appropriate media channels and platforms [51]. 

Misinformation was a prominent concern, highlighting the necessity for robust procedures to verify the accuracy and completeness of online health information [49]. Monitoring, reviewing, and updating online health information can enhance reliability [43,45]. Users should also be able to engage with content creators for clarification and report misleading information. Future research should investigate the influence of misinformation on health outcomes and explore the role of digital literacy on online HISB among PoAD. Such studies can inform the design of targeted interventions to improve digital health literacy and tackle misinformation within this community.

The use of medical jargon in online health information was viewed as a barrier by some participants, indicating the importance of providing content that is accessible and avoids technical terms [49]. Simplifying health information and utilising plain language can improve its understanding and usability.

Limited Internet connectivity and poor information retrieval abilities were identified as challenges faced by some individuals, emphasising the need for fast and reliable Internet connections and user-friendly devices [50]. Governments and service providers should prioritise the accessibility of Internet infrastructure, especially in underserved communities.

The study revealed that certain participants faced challenges in embracing online interventions due to cultural beliefs and social contexts, such as religious beliefs and conservatism. To overcome these barriers, increasing awareness about online health information and support within the specific community can be beneficial [52]. Developing health information resources that are culturally sensitive and inclusive has the potential to build trust and enhance engagement [53]. By tailoring information to align with cultural values, in addition to being inclusive of diverse perspectives, these resources can effectively bridge gaps and promote better understanding and utilisation of online health interventions.

### 4.8. Study Strengths and Limitations

This study has several strengths that contribute to its credibility and transferability. One of the key strengths is the sampling technique used to ensure that individuals of different generations from various regions across the UK were included in the study. Another strength was the use of online interviews as a data collection method. This facilitated the participation of PoAD residing in different locations across the UK, allowing us to accommodate the varied schedules of the participants. The study adopted reflexive TA as its method, which enabled in-depth inquiry into the multi-faceted drivers of the online HISBs of PoAD. This approach encourages researchers to critically explore the data, their position, and their involvement in the research process, strengthening the robustness and credibility of the set of conclusions [25].

However, the study also had some limitations. One of the limitations was the potential sampling bias due to the focus on participants who are active Internet users and have access to Internet-based devices, potentially narrowing the sample. As a result of this limitation, the findings of this study must be interpreted within this socioeconomic context, considering the intersectionality within the community. Another limitation was the possibility of social desirability bias, where participants might have provided socially favourable responses or responded in a way that aligned with the interviewer’s expectations [15]. Confidentiality of responses was assured to mitigate this bias, and honest feedback was emphasised. The absence of in-person interviews was also a limitation that might have restricted the capture of non-verbal cues and subtle nuances. The interviewer’s attentiveness addressed this limitation to vocal cues and inflexions during online interactions. Finally, the preponderance of female participants may limit the transferability of the study’s findings. Nonetheless, it does reflect broader trends in health research participation [37]. 

### 4.9. Key Implications 

The outcomes of this study offer important implications for the advancement of the Internet as a key resource for healthcare interventions and education tailored to the needs of PoAD. The prevalent use of the Internet within this community presents an opportunity to integrate the Internet into healthcare. This integration could enhance the delivery and accessibility of healthcare services.

To fully reap the benefits of online health information, it is crucial to prioritise accuracy, privacy, and security. This is particularly important in addressing the unique needs and cultural differences of PoAD. Healthcare providers and government organisations must collaborate to provide reliable, secure, and high-quality online health resources. Additionally, this partnership should involve creating culturally sensitive and relevant health initiatives that consider the diverse cultures and health beliefs present in the UK. 

Equally important is addressing challenges posed by limited health literacy, inconsistent Internet access, misinformation prevalence, and complex medical terminology. Overcoming these barriers is critical to making online health information accessible and user-friendly for PoAD and the general population. Efforts to enhance digital health literacy and provide clear, jargon-free information can significantly improve the usability of online health resources.

Finally, healthcare providers could collaborate with the PoAD to co-create culturally tailored online health resources based on the findings of this study. This collaborative effort could serve as a reliable source of health information for the PoAD in the UK and beyond. 

This approach aligns with broader efforts to ensure culturally competent healthcare delivery and health education, ultimately fostering improved health outcomes.

## 5. Conclusions

This study provides valuable insights into the online HISBs of PoAD residing in the UK. The findings reveal that PoAD actively seek health information online using various Internet-based devices, with smartphones being the predominant medium. This stresses the need for mobile-friendly health information platforms that accommodate the needs of a digitally connected population. Professional trust is another significant factor that influences the consumption of online health information among PoAD in the UK, indicating a universal respect for professional expertise. The study also reveals some barriers to online health information seeking among PoAD, such as privacy concerns, information overload, and the challenge of discerning dependable information amidst widespread misinformation. It is crucial to develop secure, accurate, and user-friendly online health resources tailored to the unique cultural and informational needs of PoAD. The role of social media in health information dissemination and the potential for misinformation underscores the importance of robust content moderation strategies and the promotion of digital literacy. Cultural influences on online HISB were also identified, suggesting a need for increased awareness and trust in online health interventions within the PoAD. By addressing these key areas, healthcare providers and policymakers can mitigate health disparities and promote equitable access to health information and services for PoAD and the wider population. 

## Figures and Tables

**Table 1:** Demographic characteristics of respondents.

Variable	*n*	%
Gender		
Female	14	67
Male	7	33
Ethnicity		
African	11	52
Afro-Caribbean	10	48
Age range (years)		
20–29	3	14
30–39	4	19
40–49	7	33
50–59	3	14
60–69	3	14
70+	1	5
Device used to Access the Internet		
Smartphone	21	100
Laptop	9	43
Desktop	8	38
Tablet	7	33
Wearables	7	33
Smart TV	6	29

## Data Availability

Data are contained within the article.
